# Comprehensive Analysis of Potential Common Pathogenic Mechanisms for COVID-19 Infection and Gastric Cancer

**DOI:** 10.1155/ancp/5106674

**Published:** 2025-03-11

**Authors:** Guiqian Zhang, Ning Wang, Shixun Ma, Yan Zhang, Pengxian Tao, Hui Cai

**Affiliations:** ^1^Otorhinolaryngology Head and Neck Surgery, The 940th Hospital of Joint Logistics Support Force of People's Liberation Army, Lanzhou, China; ^2^The First Clinical Medical College of Gansu University of Chinese Medicine (Gansu Provincial Hospital), Lanzhou, China; ^3^Key Laboratory of Molecular Diagnostics and Precision Medicine for Surgical Oncology in Gansu Province, Gansu Provincial Hospital, Lanzhou, China; ^4^General Surgery Clinical Medical Center, Gansu Provincial Hospital, Lanzhou, China; ^5^NHC Key Laboratory of Diagnosis and Therapy of Gastrointestinal Tumor, Gansu Provincial Hospital, Lanzhou, China; ^6^Cadre Ward of General Surgery Department, Gansu Provincial Hospital, Lanzhou, China

**Keywords:** COVID-19 pneumonia, differentially expressed genes, gastric cancer, hub genes, pathogenesis

## Abstract

A growing body of data suggests that the prevalence of COVID-19 pneumonia in patients with stomach cancer is much higher than in the general population. However, these mechanisms are still not fully understood. After a thorough examination of shared differentially expressed genes (DEGs) for gastric cancer (GC) and COVID-19 pneumonia, we performed functional annotation, protein–protein interaction (PPI) networks, module design, and pivot gene identification. qPCR was used to verify the expression of hub genes in GC. Finally, a pivotal gene transcription factor-gene regulatory network was created and validated. According to functional enrichment analysis, common genes are mainly enriched in biological processes such as extracellular matrix tissue and extracellular structural tissue. Finally, five genes were found to be pivotal genes in the pathogenesis of GC and COVID-19 pneumonia: BGN (biglycan), UBE2C (ubiquitin-conjugating enzymes 2C), SPP1 (secreted phosphoprotein 1), THBS2 (thrombospondin 2), and COL1A1 (type I collagen alpha 1). These shared pathways and pivotal genes could provide new insights for more mechanistic studies.

## 1. Introduction

The prevalence of gastric cancer (GC), particularly in East Asian nations, continues to be a serious worldwide health issue. GC will be the sixth most often diagnosed cancer and the third-leading cause of cancer-related deaths worldwide in 2020, with more than 1,089,103 (5.6%) diagnoses and more than 768,000 (7.7%) fatalities worldwide [1]. The regions with the highest incidence of GC are Northeast Asia, Central and South America, and Eastern Europe [2]. Although *H. pylori* infection, smoking, alcohol consumption, a high-salt diet, and physical inactivity are now widely acknowledged as independent risk factors for GC, the pathophysiology of the disease is still unknown for the majority of patients [3].

Severe acute respiratory syndrome coronavirus 2 (SARS-CoV-2) is the cause of the severe respiratory infection known as novel COVID-19 pneumonia [4, 5]. Globally, as of 12:15 p.m. CEST, June 21, 2023, there have been 768,187,096 confirmed cases of COVID-19, including 6,945,714 deaths, reported to WHO. As of June 19, 2023, a total of 13,461,344,203 vaccine doses have been administered (https://covid19.who.int/).

Angiotensin-converting enzyme 2 (ACE2) serves as the host entrance receptor for COVID-19 pneumonia, and SARS-CoV-2 targets alveolar type II epithelial cells with strong ACE2 expression [5–7]. Studies have shown that COVID-19 pneumonia is transmitted by aerosol, surface contamination, and fecal-oral routes [8].

COVID-19 pneumonia has a long incubation period, high morbidity, and is unpredictable, which makes the link between COVID-19 pneumonia and cancer due to the reduced immunity of cancer patients. Cancer patients appear to have an exacerbated condition and increased mortality once they are infected with the virus [9]. Patients who have diabetes mellitus, hypertension, chronic obstructive pulmonary disease, or cardiovascular disease are immunocompromised, which may increase their vulnerability to COVID-19 pneumonia [10, 11]. Because patients with progressive GC are immunocompromised, this is one of the group that are susceptible to COVID-19 pneumonia. This viral-induced weakening of the immune system is key to susceptibility, as GC patients already exhibit a severely weakened and altered immune system by specific cancer therapies and disease severity, putting GC patients into the ranks of increased risk. As the epidemic progresses, the incidence of patients with GC shows an increased number of severe cases [9]. Liang et al. [12] demonstrated that as compared to individuals without cancer, those who had COVID-19 pneumonia had a higher risk and frequency of significant events. COVID-19 pneumonia has a 24% mortality rate in cancer patients compared to 3% in noncancer patients [9, 13]. Patients with impaired immune systems struggle to avoid respiratory viral infections, rendering them more vulnerable to COVID-19 pneumonia. For instance, COVID-19 pneumonia led to 19% of deaths among cancer patients and other immunocompromised individuals [13]. This is sufficient data to suggest that coronaviruses contribute to the poor prognosis of cancer patients. In summary, there is also a close association between COVID-19 pneumonia and GC.

Investigating the shared transcriptional signature between GC and COVID-19 pneumonia may reveal fresh information about the shared etiology of these two illnesses. We wanted to find important genes connected to the etiology of GC concomitant with COVID-19 pneumonia in this investigation. We analyzed the dataset downloaded from the GEO database. Common differentially expressed genes (DEGs) and their roles in COVID-19 pneumonia and GC were discovered using integrated bioinformatics and enrichment analysis. The STRING (Search Tool for Retrieving Interacting Gene) database and Cytoscape software (version 3.10.1) were also used to evaluate gene modules and find pivotal genes, which was followed by the confirmation of the pivotal genes. Finally, we selected 15 crucial essential genes, confirmed them, and further developed a TF-gene regulatory network for these genes. It is anticipated that the key genes between COVID-19 pneumonia and GC discovered in this work may reveal fresh details about the biological underpinnings of these two disorders.

## 2. Methods

### 2.1. Preparation of the Dataset

It is possible to access GEO (www.ncbi.nlm.nih.gov/geo), a sizable database with gene expression information for numerous disorders, for free and in the general public [14]. The GSE103236 [15] dataset contains 10 GC tissue samples and 10 paraneoplastic tissue samples, and it is based on the Agilent-014850 Whole Human Genome Microarray 4x44K G4112F platform. The GSE118916 [16] dataset contains 15 GC tissue samples and 15 paraneoplastic tissue samples and is based on the Affymetrix Human Gene Expression Array platform. The GSE180226 dataset contains 20 COVID-19 pneumonia tissue samples and three control tissue samples and is based on the Agilent-014850 Whole Human Genome Microarray 4x44K G4112F platform. GSE164805 [17] dataset includes 10 COVID-19 pneumonia tissue samples and five control tissue samples and is based on the Agilent-085982 Arraystar human lncRNA V5 microarray platform.

### 2.2. Cell Culture

GC cells (MKN-45, AGS) and normal gastric cell (GES-1) were obtained from the Cell Resource Center of the Chinese Academy of Sciences (Shanghai, China). Cells were cultured in RPMI-1640 containing 10% fetal bovine serum, 100 units/ml penicillin and 100 μg/ml streptomycin and placed in an incubator at 37°C with 5% CO_2_.

### 2.3. RT-qPCR

Total RNA was extracted from the cells separately by Trizol method, and the purified RNA was reverse transcribed into cDNA template using a reverse transcription kit, followed by real-time quantitative PCR of the target genes under PCR light microscope, and finally the relative expression of hub genes was analyzed by 2^ΔΔct^ method and normalized with reference to GAPDH The primers for hub genes were designed and synthesized by Sangon Biotech (Shanghai, China). The primer sequences are shown in Table 1.

### 2.4. Identification of Shared DEGs Between COVID-19 Pneumonia and GC

We obtained the DEGs of GSE103236 and GSE180226 using Perl scripts, R packages “limma,” “pheatmap,” and “ggplot2,” and the shared DEGs between GSE103236 and GSE180226 datasets using R package “VennDiagram.”

### 2.5. GO and KEGG Enrichment Analysis

The R language packages “clusterProfiler,” “org.Hs.eg.db,” “enrichplot,” “ggplot2,” “circlize,” “RColorBrewer,” “dplyr,” “ggpubr,” and “ComplexHeatmap” were used for GO and KEGG enrichment analysis of the shared DEGs. The potential functions of the DEGs were analyzed. Adjusted *p* − values  < 0.05 were considered statistically significant.

### 2.6. Protein–Protein Network Construction and Module Analysis

The STRING [18] (http://string-db.org) (version 11.5) is a database that contains details on more than 14,000 species, 60 million proteins, and more than 20 billion interactions, including both direct physical interactions and indirect functional associations. STRING created protein–protein interaction (PPI) networks with interaction scores > 0.4 for frequent DEGs. PPI networks were visualized using Cytoscape software [19] (version 3.9.1), and core functional module analysis was performed using the Cytoscape plugin Molecular Complex Detection (MCODE; [20]). The parameters were set as follows: *K*-core = 2, degree cutoff = 2, and maximum depth = 100.

### 2.7. Hub Gene Identification and Analysis

Hub genes were selected via Cytoscape's plugin cytoHubba, and the final hub genes were then confirmed using degree. Coexpression networks for the hub genes were created using GeneMANIA [21] (http://genemania.org), an online program that forecasts gene connections. GO and KEGG enrichment analysis of hub genes was then performed using R.

### 2.8. Validation of Hub Genes Performance in Two Independent Cohort of Samples

We utilize the R package “limma” and “ggpubr” to validate the hub gene with the GSE118916 and GSE164805 datasets, respectively. *p* < 0.05 is considered statistically significant.

### 2.9. TF-Gene Interactions Network and TF-Gene Differential Analyze

The preTFCyto Perl script was used to build the TF-gene regulatory network, and Cytoscape was used to create and display the network. Finally, we analyzed the TF differential using R.

### 2.10. Statistical Analysis

All gene data in this study were normalized by log transformation. Differential expression of hub genes was detected by Wilcox test. Visualization of data was processed by R software (version 4.0.3). *p* < 0.05 was considered to be statistically significant.

## 3. Result

### 3.1. DEG and Shared Genes Between COVID-19 Pneumonia and GC Identification

**Figure 1:**
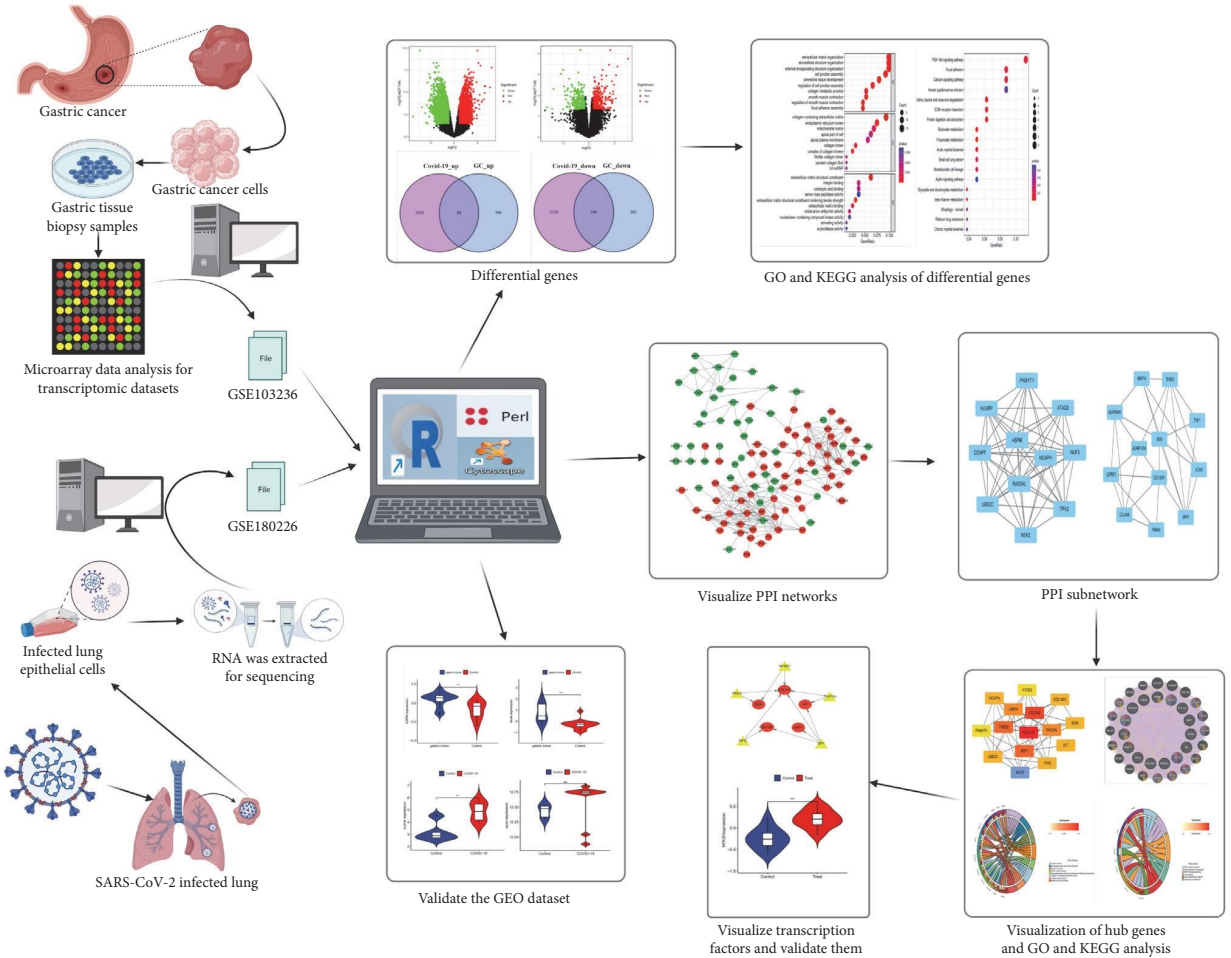
The workflow diagram of this study.

**Figure 2:**
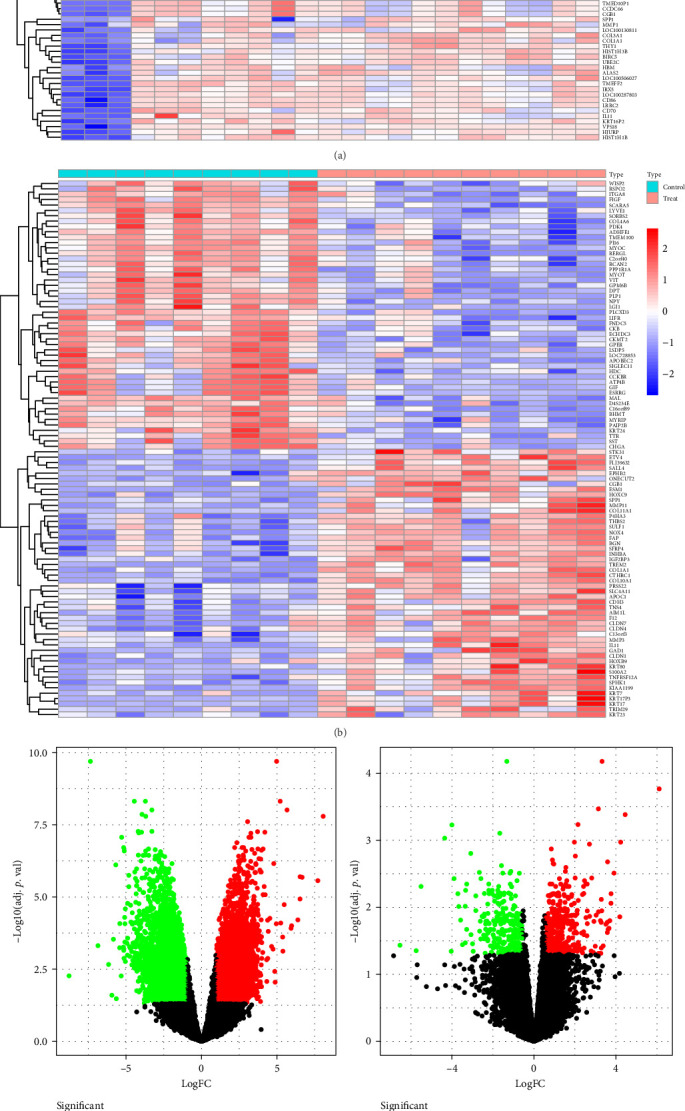
Heat map, volcano map, and Venn diagram: (A) GSE180226 heat map, (B) heat map of GSE103236, (C) GSE180226 volcano plot, and (D) GSE103236 volcano plot. Red indicates genes that are upregulated, whereas green indicates genes that are downregulated. (E, F) Venn diagram showing the upregulated and downregulated differentially expressed genes for the two datasets.

Figure 1 depicts the overall study flowchart. A total of 6404 DEGs were found in the GSE180226 dataset, of which 3176 were upregulated and 3228 were downregulated (Figure 2A,C,E). We discovered 456 DEGs using the GSE103236 dataset, including 194 downregulated genes and 262 upregulated genes (Figure 2B,D,F). Then 104 upregulated shared DEGs and 84 downregulated shared DEGs were obtained by the intersection of DEGs from the GSE180226 dataset and the GSE103236 dataset, represented by Venn diagrams (Figure 2E,F).

### 3.2. GO and KEGG Enrichment Analysis

**Figure 3:**
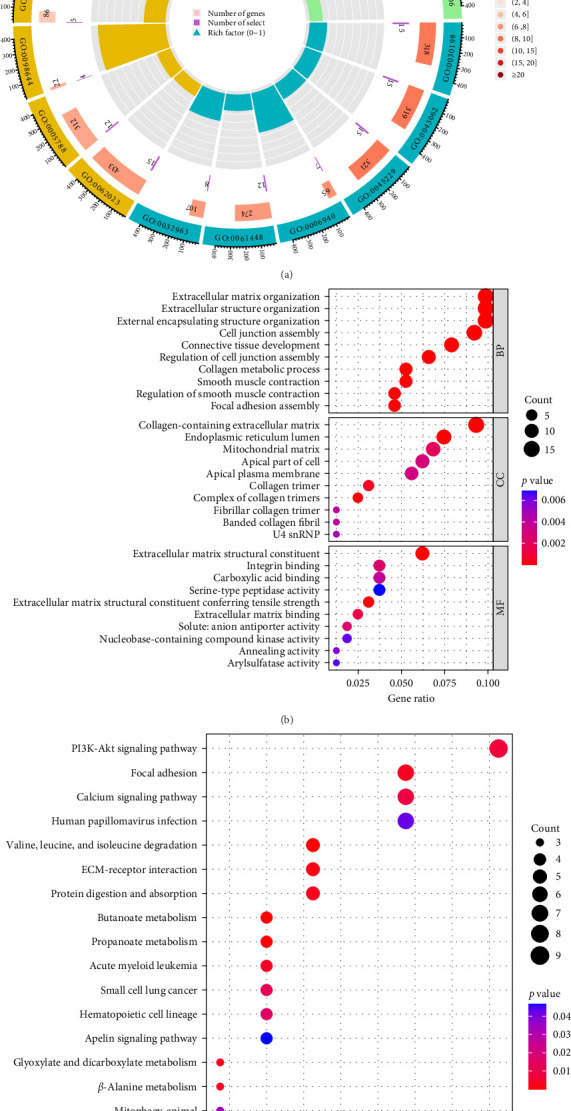
Study of common DEG enrichment using GO and KEGG: (A, B) GO enrichment analysis and (C) KEGG pathway enrichment analysis findings. The significance level was set at adjusted *p* < 0.05. DEG, differentially expressed gene.

Our study found that GO enrichment analysis regarding BPs (biological processes) shared DEGs mainly involved extracellular matrix organization and extracellular structural organization. CCs (cellular components) shared DEGs mainly involved collagen-containing extracellular matrix and endoplasmic reticulum lumen. MFs (molecular functions) shared DEGs mainly involved extracellular matrix structural constituent and integrin binding (Figure 3A,B). The top 5 significant terms in the KEGG pathway enrichment analysis were the PI3K-Akt signaling pathway, focal adhesion, calcium signaling pathway, human papillomavirus infection, and degradation of valine, leucine, and isoleucine (Figure 3C).

### 3.3. Network Study of PPIs and Submodule Analysis

**Figure 4:**
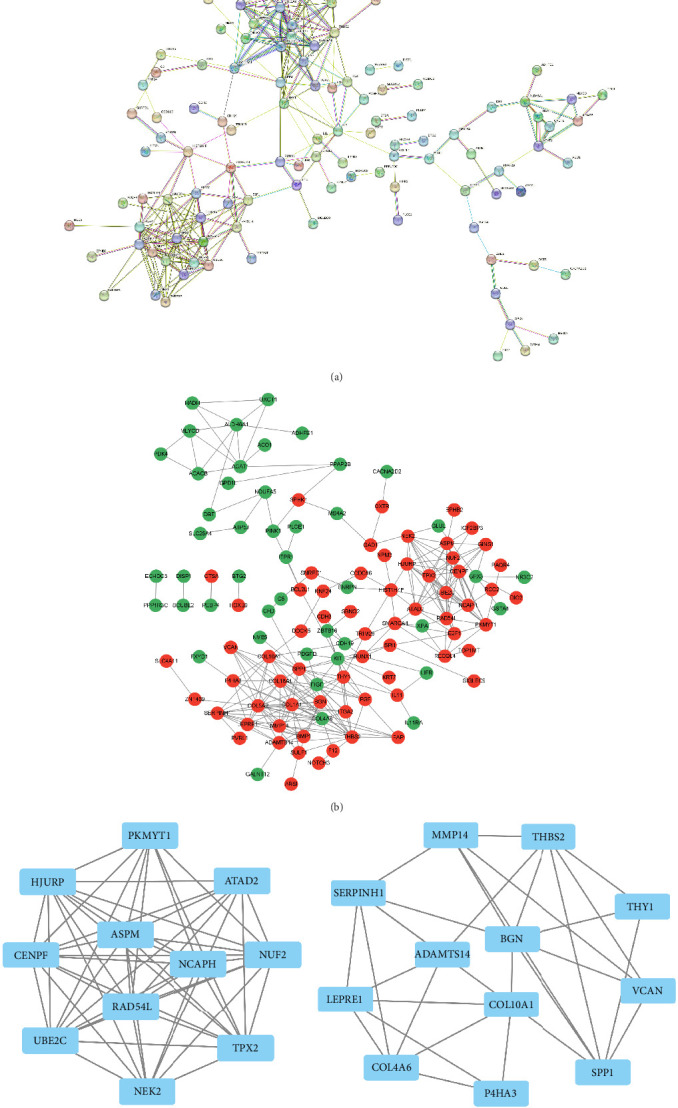
PPI network diagram: (A) PPI network diagram, (B) PPI graph after visualization, and (C, D) important gene clustering modules. The greater the gene's connectivity with other genes, the redder the color of the gene in the network. PPI, protein–protein interaction.

There were 186 nodes and 288 edges in the PPI network, and the PPI enrichment had a *p* value less than 1.0e-16 (Figure 4A). When a gene is colored red in the PPI network using the Cytoscape program, it means that there is a high degree of connection between the gene and other genes. When the color of the gene in the network is green, it indicates that the gene has low connectivity with other genes (Figure 4B). By applying Cytoscape's MCODE plug-in, two key gene modules were obtained, including 11 shared DEGs and 12 shared DEGs, respectively (Figure 4C,D).

### 3.4. Hub Gene Identification and Functional Analysis

**Figure 5:**
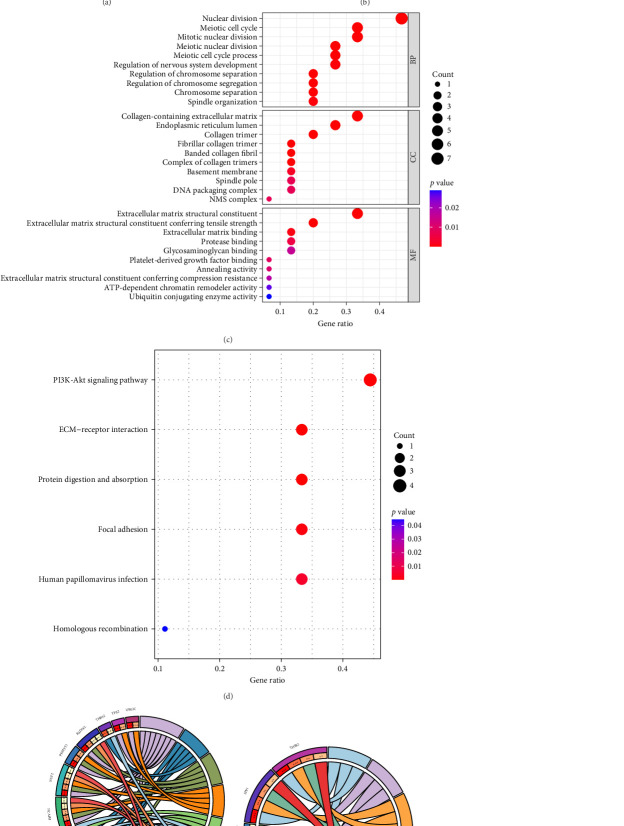
Hub gene analysis: (A) hub gene PPI; (B) hub gene coexpression network; (C, D) hub gene GO/KEGG enrichment analysis; and (E, F) circle diagram of pivotal gene GO/KEGG enrichment analysis. *⁣*^*∗∗∗*^*p* < 0.001, *⁣*^*∗∗*^*p* < 0.01, *⁣*^*∗*^*p* < 0.05.

We applied the algorithm of plugin cytoHubba to screen the top 15 hub genes, including ATAD2, COL18A1, BGN (biglycan), KIT, TPX2, NUF2, UBE2C (ubiquitin-conjugating enzymes 2C), PKMYT1, NCAPH, ASPM, COL5A2, RAD54L, SPP1 (secreted phosphoprotein 1), THBS2 (thrombospondin 2), and COL1A1 (type I collagen alpha 1) (Figure 5A). We constructed an interaction network to decipher the biological functions of these hub genes using the GeneMANIA database, where coexpression was 89.88%, shared protein domains was 6.43%, prediction was 1.92%, physical interaction was 1.75%, and colocalization was 0.01% (Figure 5B). We further explored GO and KEGG enrichment of the hub gene. The results showed that GO-related BP was mainly enriched in the nuclear division and meiotic cell cycle, CC was mainly enriched in collagen-containing extracellular matrix and endoplasmic reticulum lumen, and MF was mainly enriched in extracellular matrix structural constituent and extracellular matrix structural constituent conferring tensile strength (Figure 5C). KEGG enrichment showed that the first five pathways were PI3K-Akt signaling pathway, ECM–receptor interaction, protein digestion and absorption, focal adhesion, and human papillomavirus infection (Figure 5D). Figure 5E,F shows the circle plots of hub gene GO and KEGG enrichment analysis, respectively.

### 3.5. Hub Genes' Effectiveness Has Been Confirmed in Two Separate Cohorts of Samples

**Figure 6:**
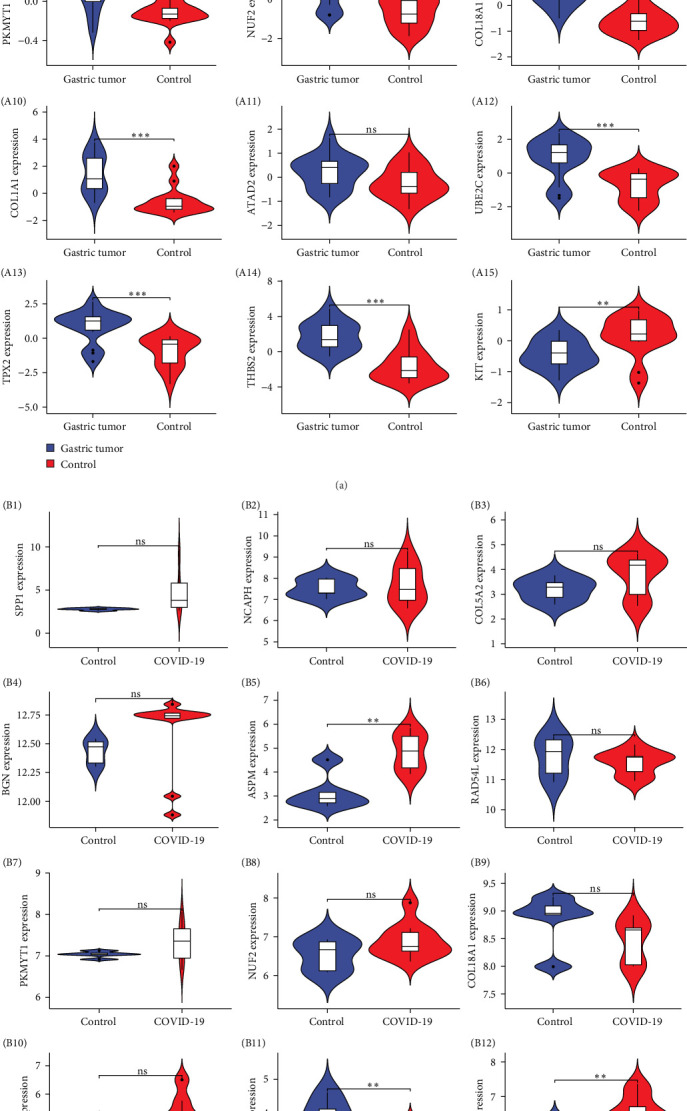
Hub gene validation: (A (A1–A15)) Validation set GSE118916; (B (B1–B15)) Validation set GSE164805. *⁣*^*∗∗∗*^*p* < 0.001, *⁣*^*∗∗*^*p* < 0.01, *⁣*^*∗*^*p* < 0.05; ns represents no significance.

Hub gene expression also showed significant differences between GC tissue samples and healthy controls (GSE118916), further indicating the reliability of sharing hub genes between the two disease states (Figure 6A). Meanwhile, hub gene expression also showed significant differences between COVID-19 peripheral blood samples and healthy controls (GSE164805) (Figure 6B). In summary, we used two separate datasets to further validate the reliability of the above hub genes, illustrating the relevance of our work.

### 3.6. TF-Gene Interaction Network and Validation of TF

**Figure 7:**
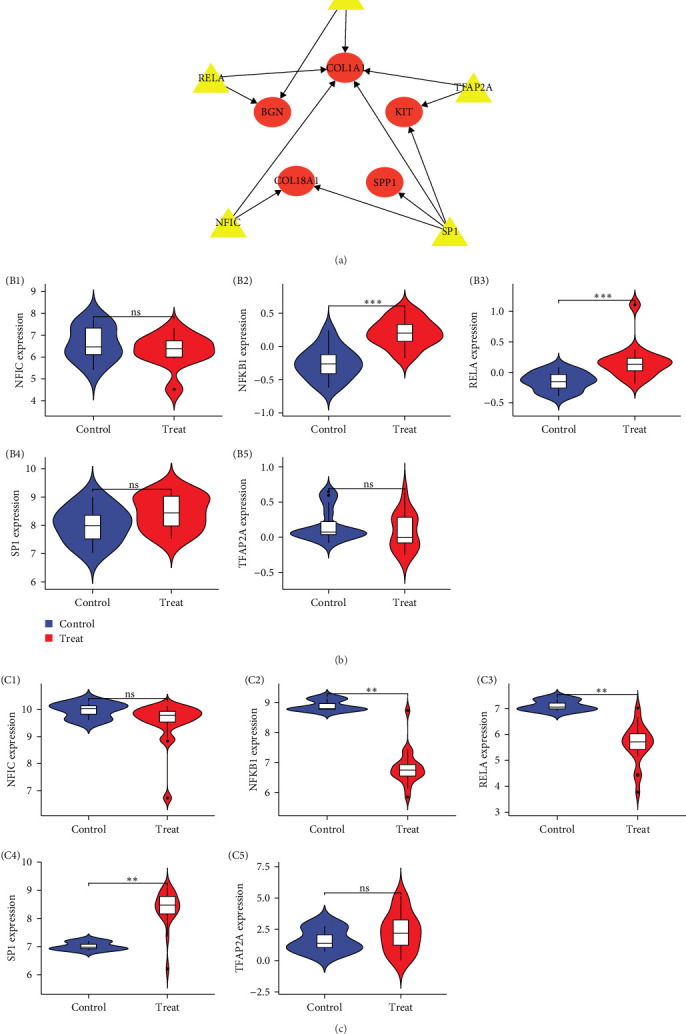
TF network and its validation: (A) TF network diagram; (B (B1–B5), C (C1–C5)) TF validation plots (GSE103236 and GSE180226 datasets). *⁣*^*∗∗∗*^*p* < 0.001, *⁣*^*∗∗*^*p* < 0.01, *⁣*^*∗*^*p* < 0.05; ns represents no significance.

The Perl script “preTFCyto” was used to predict the TFs of the five central gene interactions, and the TF-gene regulatory network was drawn and visualized by Cytoscape (Figure 7A). The network has 10 nodes, 12 edges, and 5 TFs. These TFs control multiple central genes within the network, indicating a strong degree of TF-central gene interaction. Further, we validated the TFs using the GC dataset GSE103236 and the COVID-19 pneumonia dataset GSE180226, respectively. The results showed that 50% of the TFs were meaningful in the validation of GC and COVID-19 pneumonia datasets (Figure 7B,C). This further highlights the reliability of the study.

### 3.7. Verification of the Expression of the Hub Genes in GC

**Figure 8:**
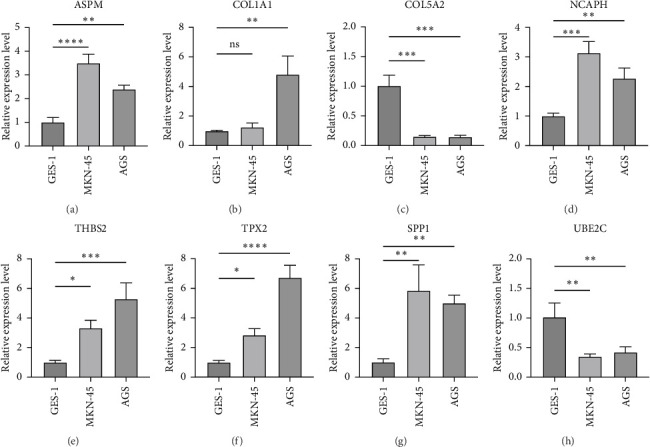
(A–H) Expression of the hub genes in gastric cancer cells. *⁣*^*∗∗∗∗*^*p* < 0.0001, *⁣*^*∗∗∗*^*p* < 0.001, *⁣*^*∗∗*^*p* < 0.01, *⁣*^*∗*^*p* < 0.05; ns represents no significance.

We selected eight hub genes, ASPM, COL1A1, COL5A2, NCAPH, THBS2, TPX2, SPP1, UBE2C, and verified them in GC cells. The results showed that ASPM, COL1A1, NCAPH, THBS2, TPX2, and SPP1 were highly expressed in GC cells, but COL5A2 and UBE2C were poorly expressed in GC cells (Figure 8).

## 4. Discussion

COVID-19 pneumonia has been present since late 2019 and its serious impact on human health has continued to date. There is evidence of a high prevalence of COVID-19 infection and a poorer prognosis in patients with GC than in the normal population [12]. The persistence of COVID-19 pneumonia has been reported to have a serious impact on the effective diagnosis and treatment of GC patients due to its presence [22]. However, studies on COVID-19 pneumonia and GC are still scarce. Therefore, we attempted to explore the common molecular biology and pathways between COVID-19 pneumonia and GC through bioinformatics approaches and tried to determine the interrelationship between COVID-19 pneumonia and GC.

Immunosuppression is a common hallmark of cancer [23]. The US National COVID-19 Cohort Collaborative consortium found that those who were fully vaccinated on immunosuppression had a 1.25–2.18 times increased risk of breakthrough infection with SARS-CoV-2 after vaccination [24]. Research has demonstrated that the median clearance duration of rhinovirus RNA and culture in individuals with severe immunosuppression resulting from hematologic malignancies or transplantation is markedly prolonged compared to those with severe immunosuppression due to autoimmune conditions or B-cell deficiencies, as well as nonseverely immunodeficient and nonimmunocompromised populations [25]. Immunocompromised GC patients are at higher risk of infection with SARS-CoV-2, but the immune deficiency that causes COVID-19 is still not fully understood.

First, we identified a large number of DEGs by exploring the GSE180226 and GSE103236 datasets. We then took the intersection of the differential genes from the two datasets and obtained 104 upregulated differential genes and 84 downregulated differential genes. Finally, we performed GO/KEGG enrichment analysis on the DEGs after intersection picking, and the results provided a powerful aid for further studies.

Further, we constructed a PPI network of common DEGs and subsequently identified 15 hub genes (ATAD2, COL18A1, BGN, KIT, TPX2, NUF2, UBE2C, PKMYT1, NCAPH, ASPM, COL5A2, RAD54L, SPP1, THBS2, and COL1A1). Previous reports have shown that five of these genes (BGN [26, 27], UBE2C [28, 29], SPP1 [30, 31], THBS2 [32, 33], and COL1A1 [34, 35]) are associated with the pathogenesis of COVID-19 pneumonia and GC. The lack of studies examining the involvement of the other hub genes in COVID-19 pneumonia or GC highlights the need for more research on these genes.

The leucine-rich small proteoglycan (SLRP) family is represented by the protein that is encoded by the BGN gene. Additionally, this protein might control innate immunity and inflammation. Foo et al. [26] showed that SARS-CoV-2 infection reshaped maternal immunity at delivery, altered the expression of cytokines associated with pregnancy complications, induced MMP7, MDK, and ESM1, and reduced BGN and CD209. Hu et al. [36] reported that BGN was overexpressed in GC tissue and promoted tumor metastasis. Huang et al. [37] reported that the SEMA3B-AS1/HMGB1/FBXW7 axis plays an inhibitory role in GC PM (peritoneal metastasis) by regulating BGN protein ubiquitination. Pinto et al. [27] showed that BGN expression in GC is associated with apoptosis, invasion, migration, and angiogenesis. In conclusion, BGN is associated with invasion and metastasis in GC.

UBE2C is a member of the ubiquitin-binding enzyme E2 family and is involved in the ubiquitin protein degradation pathway as well as playing an important role in cellular regulation [38]. UBE2C is essential for spindle assembly checkpoint and mitotic exit because of the ability of UBE2C to disrupt mitotic cyclins and securin [39]. Jin et al. [29] showed that UBE2C is highly expressed during COVID-19 pneumonia and that it may serve as a therapeutic target for COVID-19 pneumonia. Zhang et al. [28] inhibited GC cell proliferation, invasion, migration, and tumorigenesis by interfering with the expression of UBE2C. It was also determined that intestinal-type GC cells with knockdown of UBE2C had G2/M blocking. The ERK signaling pathway was stimulated and cancer cell growth was aided by UBE2C overexpression. Finally, U0126, an inhibitor of ERK signaling pathway was employed to reverse the oncogenic phenotype caused by UBE2C [28]. With chromosomal instability, intestinal-type GC may have UBE2C as a biomarker. By controlling the phosphorylation levels of Aurora-A, Wang et al. [40] demonstrated that UBE2C causes EMT through the Wnt/-catenin and PI3K/Akt signaling pathways.

A protein involved in osteoclast attachment to the mineralized bone matrix is encoded by the SPP1 gene. This protein is a cytokine that increases interferon gamma and interleukin-12 expression. SPP1 protein is expressed in COVID-19 pneumonia bronchoalveolar lavage fluid (BALF) macrophages, which activates classical monocytes in an inflammatory manner and causes neutrophils to differentiate into the proinflammatory CD274 (PD-L1) phenotype, according to research by MacDonald et al. [31]. Qian et al. [41] provided new insights into the processes and possible therapeutic targets of LNs (lymph nodes) metastasis in GC by demonstrating that abnormalities of neutrophil polarization and maturation as well as activation of the immunological checkpoint SPP1 may contribute to LN metastasis in GC. Xie et al. [30] found that tumor-specific SPP1+ macrophages drive the evolution of intratumor heterogeneous structures with tumor progression and that SPP1 can be used as a prognostic marker for patients with advanced GC and a potential therapeutic target for GC.

The protein encoded by the THBS2 gene belongs to the family of thrombospondin-responsive proteins. It is a homotrimeric glycoprotein with disulfide links that mediate interactions between cells and with the matrix. It has been demonstrated that this protein is a powerful inhibitor of tumor angiogenesis and proliferation. Genetic susceptibility has been reported to play a role in the severity of COVID-19 pneumonia, thus Cappadona et al. [32] found that genes such as THPS2 were associated with the severity of COVID-19 pneumonia. Sun et al. [42] found that THBS2 downregulation predicted poor prognosis in GC patients. Chivu-Economescu et al. [33] found that high THBS2 expression was associated with poor overall survival and immune infiltration, particularly with immunosuppressed M2 macrophages. Therefore, THBS2 may be a candidate biomarker for GC progression and prognosis and a new therapeutic target.

Type I collagen alpha 1 (COL1A1) chain encodes the pre-alpha 1 chain of type I collagen with a triple helix structure consisting of two alpha 1 chains and one alpha 2 chain. COL1A1, a member of the type I collagen family, is associated with tumor cell proliferation and invasion in a variety of cancers, including breast, lung, and kidney cancers [43–45]. Maksimowski, Williams, and Scholey [34] demonstrated that the expression of the membrane receptor ACE2 of SARS-CoV-2 was inversely correlated with genes implicated in fibrosis (COL1A1, TGFB1, and FN1) and inflammation (CCL2, IL6, and TNF). Shi et al. [35, 46] showed that high COL1A1 expression was significantly associated with the aggressiveness of GC as well as downregulation of let-7i promoted invasive metastasis of GC by targeting COL1A1. Liu et al. [47] showed that COL1A1 is a prognostic biomarker for patients with *H. pylori* (+) GC. Guo et al. [48] demonstrated that miR-133b blocks GC cell invasion and migration via the COL1A1/TGF-axis, opening up a new line of inquiry for the detection and focused therapy of GC.

The biological functions of hub genes were further explored using online databases, which revealed coexpression of 89.88%, shared protein structural domain of 6.43%, predicted of 1.92%, physical interaction of 1.75%, and colocalization of 0.01%. We then concluded that hub genes were mainly enriched in PI3K-Akt signaling pathway by KEGG enrichment analysis.

To show the reliability of sharing hub genes between the two disease states, we validated the hub genes using the GSE118916 and GSE164805 datasets. The results showed significant differences in SPP1, NCAPH, COL5A2, BGN, ASPM, RAD54L, PKMYT1, NUF2, COL18A1, COL1A1, UBE2C, TPX2, THBS2, and KIT genes.

It is well-known that TF can bind specific gene sequences to play roles in BP such as regulation of gene transcription, control of metabolism, and immune response [49]. Based on the key genes, we established a TF-gene network, and the results showed that COL1A1 had the highest interaction rate with other TF genes from the network.

Finally, we did the validation of TF-gene using GSE103236 and GSE180226 datasets. The results showed that NFKB1, RELA, and SP1 were all supportive of our previous study, while further strengthening the reliability of our study.

Of course, there are limitations in the present study. First, because there are few COVID-19 pneumonia and GC-related studies, we only selected four datasets from the GEO database for bioinformatics study and validation. Second, even though this study used a thorough bioinformatics analysis, further samples, cells, and animal trials are required to validate the findings. Third, although this study used thorough bioinformatics analysis and qPCR to verify the expression of the hub gene in GC cells, further tissue, animal testing is needed to validate these findings. Fourth, due to our limited experimental conditions, it is not possible to test the role of the hub gene in COVID-19 pneumonia. Finally, both COVID-19 and GC are inherently proinflammatory, and therefore the next step in this study will focus on the exploration of both diseases at the molecular level as well as epigenetically. As of now, however, it is unclear what the unique relationship between GC and COVID-19 is unlike other cancers and proinflammatory infections.

## 5. Conclusion

We performed enrichment and PPI network analysis and identified common DEGs for COVID-19 pneumonia and GC. This may lead to a clearer role between COVID-19 pneumonia and GC. We found a common pathogenic pathway between COVID-19 pneumonia and GC that may be mediated by specific key genes. The molecular processes of COVID-19 pneumonia and GC can be further investigated in the direction suggested by this study. This study will further verify the expression of core genes in COVID-19 models by in vitro experiments. Importantly, this study will provide help for targeted therapy of GC patients from the inflammatory molecular level.

## Figures and Tables

**Table 1:** The primer sequences.

Gene	Forward primer	Reverse primer
ASPM	5′-CCCAGACACCCGATGCCATTTG-3′	5′-TTAACCACCAAGTGAAGCCCTGTTC-3′
COL1A1	5′-AAAGATGGACTCAACGGTCTC-3′	5′-CATCGTGAGCCTTCTCTTGAG-3′
COL5A2	5′-ACGATCAAGCTAAGAACCTCAA-3′	5′-CACATTTCCATTCCGCTTAGAG-3′
NCAPH	5′-ACTACAATGTTGACACTCTGGT-3′	5′-CAAAAGTTGGAGGTGTCGTTAG-3′
THBS2	5′-AACAGCCCTGAGCCTCAGTA-3′	5′-GAAGCAGGGGTTGGATAAACAG-3′
TPX2	5′-GTCACCAAATCAGTTGACTTCC-3′	5′-AAGGATGCTTTCGTAGTTCAGA-3′
SPP1	5′-GCCGAGGTGATAGTGTGGTTTATGG-3′	5′-ATAATCTGGACTGCTTGTGGCTGTG-3′
UBE2C	5′-CAACCTTTTCAAATGGGTAGGG-3′	5′-CAGGATGTCCAGGCATATGTTA-3′

## Data Availability

The datasets used and analyzed during the current study are available from the corresponding author upon reasonable request.
